# Stochastic Network Models in Neuroscience: A Festschrift for Jack Cowan. Introduction to the Special Issue

**DOI:** 10.1186/s13408-016-0036-y

**Published:** 2016-04-04

**Authors:** Paul C. Bressloff, Bard Ermentrout, Olivier Faugeras, Peter J. Thomas

**Affiliations:** Department of Mathematics, University of Utah, 155 South 1400 East, Salt Lake City, UT 84112 USA; Department of Mathematics, University of Pittsburgh, Pittsburgh, PA 15260 USA; INRIA and LJAD, University of Nice-Sophia-Antipolis, Nice, France; Department of Mathematics, Applied Mathematics and Statistics, Case Western Reserve University, 10900 Euclid Avenue, Cleveland, OH 44106-7058 USA

## Abstract

Jack Cowan’s remarkable career has spanned, and molded, the development of neuroscience as a quantitative and mathematical discipline combining deep theoretical contributions, rigorous mathematical work and groundbreaking biological insights. The Banff International Research Station hosted a workshop in his honor, on *Stochastic Network Models of Neocortex*, July 17–24, 2014. This accompanying Festschrift celebrates Cowan’s contributions by assembling current research in stochastic phenomena in neural networks. It combines historical perspectives with new results including applications to epilepsy, path-integral methods, stochastic synchronization, higher-order correlation analysis, and pattern formation in visual cortex.

Jack Cowan’s remarkable career has spanned, and molded, the development of neuroscience as a quantitative and mathematical discipline combining deep theoretical contributions, rigorous mathematical work and groundbreaking biological insights. His achievements include an enormously successful mathematical theory for the patterning of spontaneous activity in neural networks of the human visual cortex, which underly a rich panoply of well-documented geometric visual hallucinations [11, 12, 26]. This work has also been extended to models of how cortical circuits amplify stimulus feature selectivity via spontaneous symmetry breaking mechanisms [6, 9]. One of the significant features of the hallucinations work is that it led to novel results in symmetric bifurcation theory, in particular, with regards to the so-called “shift–twist” action of the Euclidean group on $\mathbb{R}^{2}\times S^{1}$ [10]. It has also led to the development of new geometric approaches to computer vision, based on sub-Riemannian contact structures and variational problems [48, 49, 62]. Hence, the theory of hallucinations provides a nice example of how neuroscience can inspire new mathematics.

Jack Cowan’s quest to develop a self-consistent statistical treatment of the mammalian cortex began with the formulation in the mid-1960s of the firing-rate model of individual neural activity [20], which eventually led to the well-known Wilson–Cowan neural field equations [58, 59], stochastic single neuron models [44, 46, 47] and a broad class of probabilistic neural field models [14–16]. He and his students were the first to apply dynamical systems and bifurcation theory to the analysis of neural field equations [27–29]. He was also among the first to think about the developmental mechanisms of how the cerebral cortex is organized into what are known as cortical maps [57]; [23, 50, 51] and did early work on methods for solving the partial differential equations related to the propagation of electrochemical signals along neural dendrites [17], and applying neural field models to clinically important problems such as the generation of robust breathing rhythms in the mammalian brainstem [31, 32].

In addition to having a direct intellectual impact on the development of mathematical neuroscience, Jack Cowan has mentored an impressive array of students and junior colleagues. In 2014 we organized a Festschrift conference at Banff International Research Station on *Stochastic Network Models of Neocortex* (https://www.birs.ca/events/2014/5-day-workshops/14w5138) on the occasion of Jack’s 81st birthday. In tandem with the workshop we have organized this special issue of *The Journal of Mathematical Neuroscience* to highlight the history, current work, and the bright future for the mathematical modeling of stochastic cortical networks.

## Origins of Neural Field Models

In 1962, while a graduate student at MIT, Jack Cowan asked Warren McCulloch, Walter Pitts, and Norbert Wiener each what mathematical framework they thought would be best suited to studying the functioning of the human brain. According to Cowan, McCulloch thought discrete logic systems, such as the Boolean logic employed in the seminal 1943 paper on neural networks and first-order predicate logic, would be the most relevant. Pitts, in contrast, suggested moving to differential equations, so that one could exploit the tools of calculus. Norbert Wiener suggested that path-integral methods would be the best choice for studying stochastic phenomena in neural networks [22]. Cowan followed Pitts’ suggestion most closely and devoted much attention to formulating integro-differential equations to represent neural activity. Noting an analogy between predator–prey systems and excitatory–inhibitory interactions in the central nervous system, Cowan put excitatory populations in the role of prey, with inhibitory populations taking the role of predators. This insight led to the first application of qualitative dynamical systems analysis to neural field equations. Much later, he would realize the significance of Wiener’s suggestion as he ultimately developed path-integral machinery for studying stochastic neural field equations with Ohiro, Buice, Chow, Neuman, and others [14–16, 24, 25, 45].

The paper by Cowan, Neuman and van Drongelen in the present collection [24] reviews the historical development of the mean field and stochastic Wilson–Cowan equations, and describes new results related to the statistical behavior of cortex under resting conditions and in response to weak and strong stimulation. The paper reviews experimental studies showing that (1) resting cortex exhibits sparse spontaneous activity that is temporally and spatially correlated; (2) when resting cortex is disturbed by a weak stimulus a wave of cortical activity propagates from the stimulation site at a large velocity (circa 0.3 mm/msec), while decrementing exponentially [41, 42]; and (3) when resting cortex is disturbed by a stronger stimulus a longer lived active state is evoked that propagates more slowly (circa 0.1 mm/msec). The authors argue that the resting cortex and the weakly driven cortex exist in a fluctuation driven regime, the statistical structure of which corresponds to a system near a continuous phase transition in the universality class of directed percolation, close to a Bogdanov–Takens bifurcation; and, moreover, that the essential features of the activity near this transition are captured by a stochastic Wilson–Cowan equation based on an underlying two-state model [4, 55]. In contrast, they argue that the strongly driven cortex enters a mean-driven state that is well described by the original mean-field equations.

## Applications to Epilepsy

Cowan’s earliest attempts at a statistical treatment of cortical activity relied on an analogy with the predator–prey equations, with inhibitory cells playing the role of the predator and excitatory cells playing the role of prey. The quadratic nonlinearities in the earliest models (e.g. due to Kerner [37]) were equivalent, under a change of variables, to a Hamiltonian system, meaning they could not possess asymptotically stable attractors such as attracting fixed points or asymptotically stable limit cycles. A crucial innovation was to exploit dissipative dynamics, which Cowan and Wilson accomplished by introducing a sigmoidal activation function as a canonical saturating response function [58]. However, the sigmoid is a monotonically increasing function of its input, so it cannot capture nonmonotonic response behavior, for instance cessation of firing under increasing synaptic drive due, also known as depolarization block. But just such a mechanism has been proposed to underly propagation of pathological firing activity in some forms of epilepsy (inhibitory interneurons driven into depolarization block leading to failure of inhibitory veto) as well as spreading depression [56]. In the present issue, Meijer et al. [40] augment the Wilson–Cowan framework by replacing the classic monotonic activation function with a bell-shaped curve that reflects decreasing response to sufficiently large stimulation. They perform a bifurcation analysis of the Wilson–Cowan model with such a Gaussian activation function. This modification of the original equations leads to a new stable equilibrium with elevated activity in the excitatory population, and suppressed activity in the inhibitory population. The authors demonstrate numerically that these equations can sustain propagating wave solutions which they compare with their own experimental observations of epileptiform activity.

The local Wilson–Cowan equations [58] and the spatially extended equations [59] can produce saddle-node, Hopf, Turing, and interacting Turing–Hopf bifurcations. Negahbani et al. [43] augment the W-C field equations with small additive white noise perturbation of the *E* and *I* population activities, and study the resulting behavior near each type of bifurcation using numerical simulation and linear noise analysis. They demonstrate critical slowing down, growth of long-range spatial correlations, and noise enhanced pattern formation. For comparison, they include experimental evidence of corresponding phenomena preceding “seizure-like events” in murine hippocampal slices treated with carbachol in a low-magnesium artificial cerebrospinal fluid preparation. By leveraging the relative tractability of the 1D spatially extended Wilson–Cowan equations they propose a novel tool for seizure prediction based on changes in cortical activity at low spatial and temporal frequencies.

## Path-Integral Methods

Modern advances in the theory of stochastic activity in neuronal networks have relied heavily on path-integral methods. Wiener originated path-integral methods for studying stochastic processes in the 1920s, and they have found wide application in quantum field theory and statistical mechanics. Cowan and Butz formulated an early diagrammatic approach for solving the cable equation in arbitrary branched dendritic trees with constant conductivity [17], and Cowan and Ohira developed a path-integral approach to stochastic neural dynamics circa 1993 [45]. But a clear formulation of the path-integral approach to stochastic neural dynamics awaited the work of Buice, Cowan and Chow [14–16]. In the present issue Chow and Buice [19] provide a tutorial introduction to path-integral methods specifically tailored to the case of stochastic differential equations arising in neural dynamics. The article begins with the generating function formalism from elementary probability theory before working up to the path-integral formalism. The article carefully presents analytically solvable cases, such as the Ornstein–Uhlenbeck process, as a foundation for perturbative approaches to nonlinear dynamics. They emphasize practical aspects such as how to use the response function method to obtain moments of the density, and go on to address moment generating functionals for ensembles of trajectories, functional integrals for SDEs, and Feynman diagrams, including semiclassical approximation via loop expansion.

Bressloff’s contribution to the special issue [5] develops the path-integral framework further by considering a hybrid stochastic network in which neuronal activity works as a discrete conditional Markov process influenced by slowly varying conditionally deterministic synaptic activations. The resulting piecewise deterministic (or hybrid) Markov process enjoys a separation of timescales; exploiting the small parameter allows for derivation of a variational principle, analysis of most likely escape paths from a metastable state, rigorous derivation of a diffusion approximation near a stable fixed point, analysis of noise enhanced Turing-type pattern formation, and a correction to the voltage-based mean field equations by way of a loop expansion. In all, the paper illustrates how the path-integral representation provides a unifying framework for a plethora of asymptotic perturbation methods applicable to a broad class of hybrid stochastic neural network models.

## Stochastic Synchronization

Synchronization and entrainment of nonlinear oscillators is an aspect of neuronal dynamics playing an important role in both normal and pathological activity. Rhythms play a constructive role in respiration, for instance [31, 32]. In the present issue Verduzco-Flores [54] studies stochastic synchronization of uncoupled oscillators with type I phase resetting curves as an approach to understanding coordinated firing of Purkinje cells, implicated as part of the cerebellar circuitry impacting motor control and motor learning. The author overcomes the technical challenge posed by coordinated excitatory and lagged inhibitory input from climbing fibers and molecular-level interneurons through a succession of analytic and numerical refinements of an effective phase response curve model. Stochastic synchronization has a long history in neuroscience. Early examples involving nerve cells driven by fluctuating inputs included Bryant and Segundo’s white noise experiments on *Aplysia* motor neurons [13] and Mainen and Sejnowski’s demonstration of reliable spike timing in mammalian cortical neurons [39]. Cowan and collaborators helped provide an early explanation for these phenomena in terms of nonlinear oscillator entrainment and resonance [34], and established conditions guaranteeing phase locking of neuronal oscillators [33]. Indeed the existence of limit cycle oscillations was an important feature of the original deterministic Wilson–Cowan network [27, 58, 59] which provided an early explanation for the oscillations observed in EEG recordings; synchronization in Wilson–Cowan networks has been widely investigated [18].

## Beyond Mean-Field Correlation Analysis

The contribution by Fasoli et al. [30] uses a non-spiking voltage model with sigmoidal activation function, driven by both deterministic and additive white noise currents, for a rigorous analysis of correlated activity. The authors develop a novel formalism for evaluating the cross-correlation structure of a finite-size recurrently connected network. They incorporate three sources of variability (in the initial voltages, in the additive white noise current, and in the synaptic weight distribution) and five small parameters: the noise intensity, standard deviation of the initial voltage ensemble, standard deviation of the synaptic weight ensemble, amplitude of the time-varying component of the synaptic weights, and amplitude of the time-varying component of the input current. By expanding in these small parameters, and assuming Gaussian distributions for each source of variability, the authors obtain analytic expressions for the *n*-fold covariance of the voltages of each cell in the network, as well as (asymptotically) the covariances of the firing rates. The article demonstrates how one can relate anatomical and functional connectivity analytically, and fully develops the cases of certain regular graphs, specifically networks with block-circulant and hypercube topologies. Interestingly, they find that pairwise correlations in the network decrease when the constant (DC) component of the driving current is increased to large values, because saturation of the sigmoidal activation function weakens the effective connectivity when the driving current is large. In addition, Fasoli et al. find that for certain parameter values, the neurons can become almost perfectly correlated even if the sources of randomness are independent.

Leen and Shea-Brown [38] further extend the analysis of *n*-point correlations induced by common noise inputs by studying the emergence of beyond-pairwise correlations in two spiking neuron models: the exponential integrate-and-fire (EIF) model with cells driven by partially correlated white noise currents, and a linear–nonlinear (LNL) spiking model, a doubly stochastic point process derived from the EIF. The binned spike trains obtained from these models exhibit stronger higher-order spike count correlations than could be predicted under a pairwise maximum entropy Ansatz from the first- and second-order statistics alone. The authors then relate these beyond-pairwise correlations to those obtained in more abstract “dichotomous Gaussian” (DG) models [3, 61] both numerically and semi-analytically. Although the authors’ approach neglects physiological effects such as a neuron’s refractory period (which played a key role in the original Wilson–Cowan derivation), it nevertheless helps explain why the simple DG model succeeds in capturing higher-order correlations observed in empirical data from some populations of spiking neurons.

## Pattern Formation in Visual Cortex

Beginning in the 1970s Cowan and his students pioneered the application of equivariant bifurcation theory to pattern formation in cortical networks. First-principles explanation of a broad class of geometric visual hallucination patterns began with Cowan’s work with Ermentrout [26] and later extended to include anatomical details such as long-range anisotropic, orientation-specific connectivity [7, 10–12, 21]. Similar methods yielded results on the formation of connectivity patterns underlying phenomena such as orientation preference, ocular dominance, and spatial frequency maps in primary visual cortex (area V1) [2, 6, 8, 9, 50–52]. Two contributions in this collection report on continued advances in understanding pattern formation in cortical activity and connectivity patterns. The paper by Afgoustidis [1] builds on work by Wolf, Geisel, Kasschube and others [35, 36, 60] which showed a surprising statistical regularity in both imaging studies of orientation preference maps and phenomenological models for map formation in the presence of symmetry constraints, namely, that the typical density of orientation preference singularities (“pinwheels”) and the hypercolumn area are in a constant ratio of *π* across species. Wolf and Geisels’ analysis, like that in Cowan’s papers, were developed assuming the symmetries of the Euclidean plane. Afgoustidis extends the analysis for the specification of “typical” orientation patterns to symmetric manifolds $\mathcal{M}$ of constant curvature, namely the sphere and the hyperbolic plane as well as Euclidean space. He generalizes the key elements of Wolf and Geisel’s framework: smooth Gaussian random fields, invariance with respect to an appropriate Lie group of transformations of $\mathcal{M}$, and monochromaticity, i.e. composition of orientation preference maps via superposition of (uniformly, randomly distributed) “plane waves” with a common wavelength. This last element is interpreted in terms of the irreducible factors in the Plancherel decomposition of the Hilbert space of square-integrable functions on the Euclidean plane, the sphere, and the hyperbolic plane, respectively.

Cowan and colleagues studied spontaneous pattern formation using a model in which the cortical activity was represented as a function $a(\mathbf{r},\phi)$ depending simultaneously on cortical (or retinotopic) location and preferred orientation. Veltz et al. [53] challenge and extend this work by incorporating a discrete lattice symmetry into the representation of the cortical distribution of orientation preference, i.e. they study patterns in the activity $a(\mathbf{r})$ when a lattice-periodic orientation map $\phi(\mathbf{r})$ is imposed. They investigate a model in which isotropic local coupling is perturbed by weak anisotropic lateral coupling in order to understand how long-range connections with discrete lattice symmetry would affect the stability of different patterns of spontaneous activity (hallucination patterns). Their analysis shows that the possible periodic lattices of pinwheels (orientation preference singularities) is a subset of the wallpaper groups of Euclidean symmetries, and that the simplest spontaneously bifurcating dynamics generated by these networks are determined by the perturbation of invariant tori.
